# Pyloric Atresia in Association with Multiple Colonic Atresias in a Neonate: An Unreported Association

**Published:** 2012-01-01

**Authors:** Vijay C Pujar, Santosh Kurbet, Deepak K Kaltar

**Affiliations:** Department of Paediatric Surgery, J.N.Medical College Belgaum Karnataka, India

**Keywords:** Congenital pyloric atresia, Multiple colonic atresias

## Abstract

Pyloric atresia is rare cause of gastrointestinal obstruction in neonates and usually occurs as an isolated anomaly. They have been associated with multiple small bowel and colonic atresias but not reported in association with isolated multiple colonic atresias. A case of pyloric atresia occurring in association with multiple colonic atresias is being reported here.

## INTRODUCTION

Pyloric atresia is rare cause of gastrointestinal (GI) obstruction in a neonate and usually occurs as an isolated abnormality [1]. Multiple colonic atresias are another rare cause of GI obstruction in neonates. They are not known to occur together. We encountered a neonate with presence of these two anomalies. No similar association has been reported in literature to the best of our knowledge.

## CASE REPORT

A 1-day-old (weight 2.5kg) male neonate, born out of non consanguineous marriage by full term vaginal delivery was referred to us with distension of abdomen and failure to pass meconium since birth. No antenatal details were available. On examination neonate was hemodynamically stable. Abdomen was grossly distended with stretched shiny skin. Erythema was present in the periumbilical region indicating underlying peritonitis.

Both the scrotal sacs were filled with fluid. Routine hematology investigations were within normal limits. Erect X-ray abdomen revealed single air fluid level in the Lt. Upper quadrant with rest of abdomen having ground glass appearance with specks of calcification seen in left flank area (Fig. 1).

**Figure:**
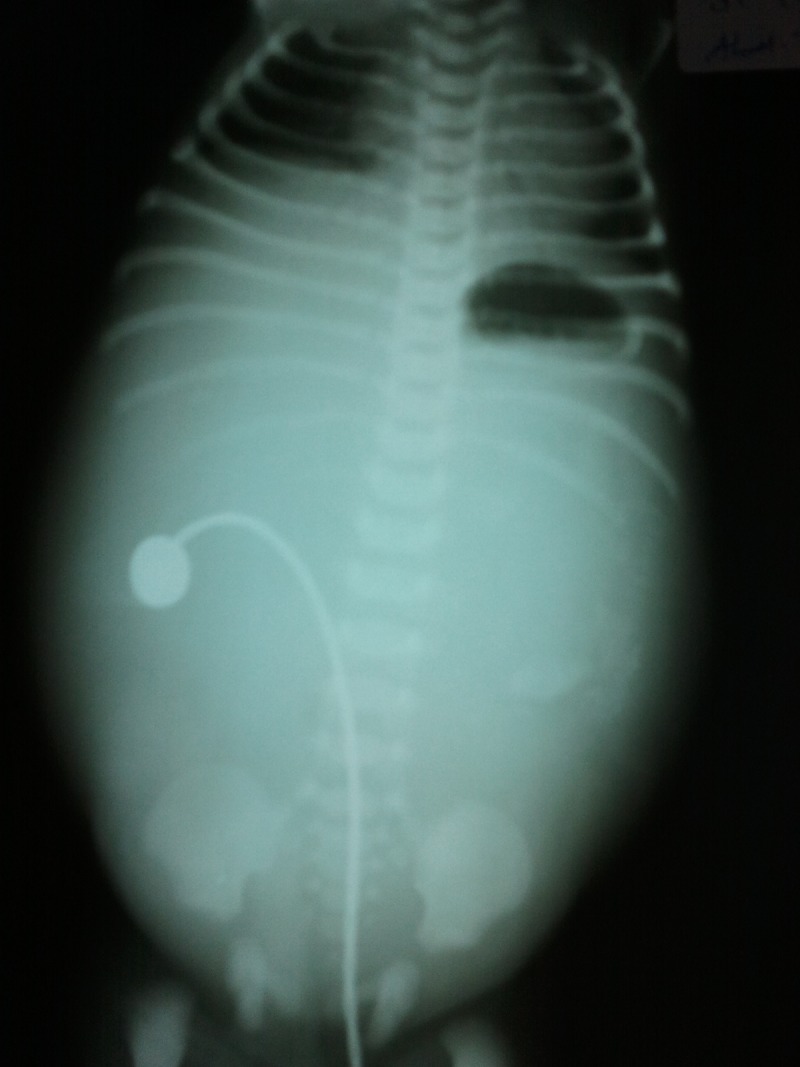
Figure 1: Radiograph showing single air bubble with calcifications in left flank

Baby was resuscitated with nasogastric tube drainage, IV fluids and antibiotics. Provisional diagnosis of upper gastro-intestinal obstruction with associated peritonitis was made. At operation the peritoneal cavity was filled with meconium stained fluid. Small bowel, caecum and part of ascending colon were dilated. A large perforation was noticed on the anti-mesenteric border of the ascending colon. Multiple colonic atresias were present distal to the perforation site, near the hepatic flexure and in transverse colon (intra luminal webs Type I) (Fig. 2).

**Figure:**
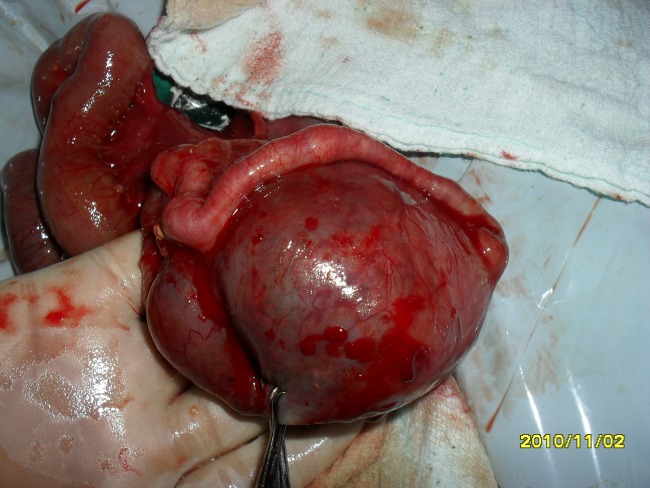
Figure 2: Type I transverse colon atresia

Two more Type II atresias were noticed near the recto sigmoid junction. Resection of atretic segments with end to back anastomosis at three sites and removal of intra luminal web were done along with covering ileostomy.

Gastrotomy done proximal to the pylorus revealed type I A pyloric atresia (complete Web). There was no discon­tinuity at pylorus. Resection of pyloric web with pyloroplasty (Heineke-Mikulicz) was done. Gastrostomy was done by putting 8-Fr Malecot’s catheter. Bilateral flank drains were inserted.

Post operatively baby required ventilator support and frequent assessment for fluid and electrolyte levels. Feeds were started through gastrostomy tube on 10th post operative and drain removed on 10th postoperative day. The patient is doing well and on follow up for ileostomy reversal.

## DISCUSSION

Congenital pyloric atresia (CPA) is a very rare condition that was first described by Calder in 1749.Its incidence is approximately 1 in 100,000 newborns and constitutes about 1% of all intestinal atresias [1,2].

Anatomically, Pyloric Atresia is divided into three different anatomical varieties: type A is a prepyloric mem¬brane, type B a solid core of tissue replacing the pyloric canal, and type C an atretic pylorus with a gap between stomach and duodenum [3].

The diagnosis of pyloric atresia may be suspected from plain abdominal films consistent with a dilated stomach with the typical ‘single bubble’ appearance as seen in our case [4]. Ultrasonography, said to be specific for this condition was not performed in our case.

Pyloric atresia may occur as an isolated condition or associated with other abnormalities, the most common being junctional epidermolysis bullosa (EB), a rare autosomal recessive disorder affecting the skin and mucosa [3-6]. However, its association with multiple colonic atresias is not reported to date; though it has been re¬ported in association with multiple small bowel and colonic atresias.

Colonic and pyloric atresias have a different embryological basis and different period of occurrence. Colonic atresia is a late event usually occurs beyond 24 weeks of gestation. Whereas, pyloric atresia is thought to result from developmental arrest between the 5th and 12th weeks of intrauterine life [2,7].

The treatment of CPA is surgical. For pyloric diaphragm or pyloric atresia without a gap, the treatment is excision of the diaphragm and Heineke-Mikulicz pyloroplasty. Intraoperatively it is important to locate the site of obstruction especially in those with a diaphragm, and to obviate missing a windsock diaphragm, a catheter should be passed distally via a small gastrostomy. This is also of importance in case there is more than one diaphragm. For those with pyloric atresia with a gap, if the gap is short, they should be treated with a Finny or Heineke-Mickulicz pyloroplasty, but if the gap is long then a gastroduodenostomy becomes the treatment of choice [7].

In conclusion, our case is unique because of occurrence of both anomalies in the same patient. No similar report was found in the literature.

## Footnotes

**Source of Support:** Nil

**Conflict of Interest:** None declared

